# Supplementary material for: Validation of the youth version of the Alimetry® Gut-Brain Wellbeing Survey: a mental health scale for young people with chronic gastroduodenal symptoms
Source: World J Pediatr. 2026 Apr 29;22(4):465–76. doi: 10.1007/s12519-026-01027-4 (PMC13221431; doi:10.1007/s12519-026-01027-4)
Supplement: Supplementary file 1 — Supplementary file1 (PDF 122 KB) [file 12519_2026_1027_MOESM1_ESM.pdf]

Alimetry® Gut-Brain Wellbeing Survey- Youth Version (AGBW-Y)

The following 10 questions will ask you about how you have been feeling over the past 2 weeks.

Sometimes, how your stomach feels can affect your mood and your mood can also affect your stomach. It’s almost like your stomach and brain are talking to each other. Answering these questions will help your doctor understand how you are feeling and make a plan to help you feel better.

Your answers are private and will only be seen by your doctor. These questions can’t diagnose you with anything, they're just to help your doctor understand you better.

- ☐ Skip wellbeing survey
  If you want to, you can tell us why you don't feel like answering these questions
  ____
- ☐ Continue

1. Over the last 2 weeks, how often did you **feel not as excited** about things that usually make you happy?

None of the time	A little of the time	Some of the time	Most of the time	All of the time
☐	☐	☐	☐	☐

2. Over the last 2 weeks, how often have you felt **sad, down, or unhappy**?

None of the time	A little of the time	Some of the time	Most of the time	All of the time
☐	☐	☐	☐	☐

3. Over the last 2 weeks, how often have you felt really **tired, like you had no energy,** even when there wasn’t a good reason for it?

None of the time	A little of the time	Some of the time	Most of the time	All of the time
☐	☐	☐	☐	☐

4. Over the last 2 weeks, how often did you find it **hard to think or pay attention**?

None of the time	A little of the time	Some of the time	Most of the time	All of the time
☐	☐	☐	☐	☐

© Alimetry Ltd., 2025. Please contact stefan@alimetry.com for any licensing requests

5. Over the last 2 weeks, how often did you feel like you were having **a hard time handling** the problems or troubles in your life?

None of the time	A little of the time	Some of the time	Most of the time	All of the time
☐	☐	☐	☐	☐

6. Over the last 2 weeks, how often did you feel like you **were not in control** of the important things in your life?

None of the time	A little of the time	Some of the time	Most of the time	All of the time
☐	☐	☐	☐	☐

7. Over the last 2 weeks, how often did you feel like things were **not going well** for you in your life?

None of the time	A little of the time	Some of the time	Most of the time	All of the time
☐	☐	☐	☐	☐

8. Over the last 2 weeks, how often did you feel **tense, nervous, or like you just couldn't relax**?

None of the time	A little of the time	Some of the time	Most of the time	All of the time
☐	☐	☐	☐	☐

9. Over the last 2 weeks, how often were you **worried** about things in your life?

None of the time	A little of the time	Some of the time	Most of the time	All of the time
☐	☐	☐	☐	☐

10. Over the last 2 weeks, how often did you feel **scared or afraid,** like something bad might happen, even when there wasn't a real reason to feel that way?

None of the time	A little of the time	Some of the time	Most of the time	All of the time
☐	☐	☐	☐	☐

If you have anything else you want your doctor to know about your answers, you can write below. But you don't have to.

© Alimetry Ltd., 2025. Please contact stefan@alimetry.com for any licensing requests
