# Supplementary material for: Validation of the youth version of the Alimetry® Gut-Brain Wellbeing Survey: a mental health scale for young people with chronic gastroduodenal symptoms
Source: World J Pediatr. 2026 Apr 29;22(4):465–76. doi: 10.1007/s12519-026-01027-4 (PMC13221431; doi:10.1007/s12519-026-01027-4)
Supplement: Supplementary file 2 — Supplementary file2 (DOCX 10 KB) [file 12519_2026_1027_MOESM2_ESM.docx]

**Table S1.** Percentile ranks for the total and subscale scores for the Alimetry Gut-Brain Wellbeing Survey-Youth Version (AGBW-Y) in a sample of n=128 young people aged 12-17 years old with chronic gastrodudoenal symptoms.

| **Raw Score** | **Percentile Ranks** | | | |
| --- | --- | --- | --- | --- |
|  | **Total Score** | **Depression Subscale** | **Stress Subscale** | **Anxiety Subscale** |
| **0** | 0 | 0 | 0 | 0 |
| **1** | 2 | 2 | 7 | 6 |
| **2** | 3 | 4 | 10 | 9 |
| **3** | 4 | 7 | 14 | 17 |
| **4** | 4 | 12 | 22 | 24 |
| **5** | 5 | 16 | 30 | 37 |
| **6** | 5 | 23 | 41 | 42 |
| **7** | 6 | 34 | 54 | 57 |
| **8** | 9 | 41 | 60 | 66 |
| **9** | 10 | 53 | 72 | 76 |
| **10** | 13 | 63 | 80 | 85 |
| **11** | 17 | 77 | 87 | 90 |
| **12** | 20 | 82 | 91 | 94 |
| **13** | 23 | 89 |  |  |
| **14** | 25 | 93 |  |  |
| **15** | 27 | 95 |  |  |
| **16** | 30 | 98 |  |  |
| **17** | 33 |  |  |  |
| **18** | 39 |  |  |  |
| **19** | 43 |  |  |  |
| **20** | 45 |  |  |  |
| **21** | 47 |  |  |  |
| **22** | 52 |  |  |  |
| **23** | 55 |  |  |  |
| **24** | 59 |  |  |  |
| **25** | 65 |  |  |  |
| **26** | 68 |  |  |  |
| **27** | 71 |  |  |  |
| **28** | 74 |  |  |  |
| **29** | 78 |  |  |  |
| **30** | 81 |  |  |  |
| **31** | 87 |  |  |  |
| **32** | 89 |  |  |  |
| **33** | 92 |  |  |  |
| **34** | 93 |  |  |  |
| **35** | 94 |  |  |  |
| **36** | 95 |  |  |  |
| **37** | 97 |  |  |  |
| **38** | 98 |  |  |  |
| **39** | 98 |  |  |  |
| **40** | 99 |  |  |  |
